# Cytotoxicity and Apoptosis Effects of Curcumin Analogue (2E,6E)-2,6-Bis(2,3-Dimethoxybenzylidine) Cyclohexanone (DMCH) on Human Colon Cancer Cells HT29 and SW620 In Vitro

**DOI:** 10.3390/molecules26051261

**Published:** 2021-02-26

**Authors:** Nurul Fattin Che Rahim, Yazmin Hussin, Muhammad Nazirul Mubin Aziz, Nurul Elyani Mohamad, Swee Keong Yeap, Mas Jaffri Masarudin, Rasedee Abdullah, Muhammad Nadeem Akhtar, Noorjahan Banu Alitheen

**Affiliations:** 1 Department of Cell and Molecular Biology, Faculty of Biotechnology and Biomolecular Sciences, University Putra Malaysia, UPM, Serdang 43400, Selangor, Malaysia; nurulfattincherahim@gmail.com (N.F.C.R.); yazminh93@gmail.com (Y.H.); muhammadnazirulmubin@gmail.com (M.N.M.A.); elyani.mohamad@gmail.com (N.E.M.); masjaffri@upm.edu.my (M.J.M.); 2 Biotechnology Research Institute, University Malaysia Sabah, Kota Kinabalu 88400, Sabah, Malaysia; 3 China-ASEAN College of Marine Sciences, Xiamen University Malaysia, Sepang 43900, Selangor, Malaysia; skyeapxmu@gmail.com; 4 UPM-MAKNA Cancer Research Laboratory, Institute of Bioscience, University Putra Malaysia, Serdang 43400, Selangor, Malaysia; 5 Department of Veterinary Pathology and Microbiology, Faculty of Veterinary Medicine, University Putra Malaysia, UPM, Serdang 43400, Selangor, Malaysia; rasedee@upm.edu.my; 6 Faculty of Industrial Sciences & Technology, University Malaysia Pahang, Lebuhraya Tun Razak, Kuantan 26300, Pahang, Malaysia; nadeemump@gmail.com

**Keywords:** colon cancer, curcumin analogue, DMCH, cytotoxic, apoptosis, cell cycle

## Abstract

Colorectal cancer (CRC) is the third most common type of cancer worldwide and a leading cause of cancer death. According to the Malaysian National Cancer Registry Report 2012–2016, colorectal cancer was the second most common cancer in Malaysia after breast cancer. Recent treatments for colon cancer cases have caused side effects and recurrence in patients. One of the alternative ways to fight cancer is by using natural products. Curcumin is a compound of the rhizomes of *Curcuma longa* that possesses a broad range of pharmacological activities. Curcumin has been studied for decades but due to its low bioavailability, its usage as a therapeutic agent has been compromised. This has led to the development of a chemically synthesized curcuminoid analogue, (2E,6E)-2,6-bis(2,3-dimethoxybenzylidine) cyclohexanone (DMCH), to overcome the drawbacks. This study aims to examine the potential of DMCH for cytotoxicity, apoptosis induction, and activation of apoptosis-related proteins on the colon cancer cell lines HT29 and SW620. The cytotoxic activity of DMCH was evaluated using the [3-(4,5-dimethylthiazol-2-yl)-2,5-diphenyltetrazolium bromide] (MTT) cell viability assay on both of the cell lines, HT29 and SW620. To determine the mode of cell death, an acridine orange/propidium iodide (AO/PI) assay was conducted, followed by Annexin V/FITC, cell cycle analysis, and JC-1 assay using a flow cytometer. A proteome profiler angiogenesis assay was conducted to determine the protein expression. The inhibitory concentration (IC_50_) of DMCH in SW620 and HT29 was 7.50 ± 1.19 and 9.80 ± 0.55 µg/mL, respectively. The treated cells displayed morphological features characteristic of apoptosis. The flow cytometry analysis confirmed that DMCH induced apoptosis as shown by an increase in the sub-G0/G1 population and an increase in the early apoptosis and late apoptosis populations compared with untreated cells. A higher number of apoptotic cells were observed on treated SW620 cells as compared to HT29 cells. Human apoptosis proteome profiler analysis revealed upregulation of Bax and Bad proteins and downregulation of Livin proteins in both the HT29 and SW620 cell lines. Collectively, DMCH induced cell death via apoptosis, and the effect was more pronounced on SW620 metastatic colon cancer cells, suggesting its potential effects as an antimetastatic agent targeting colon cancer cells.

## 1. Introduction

**Figure 1:**
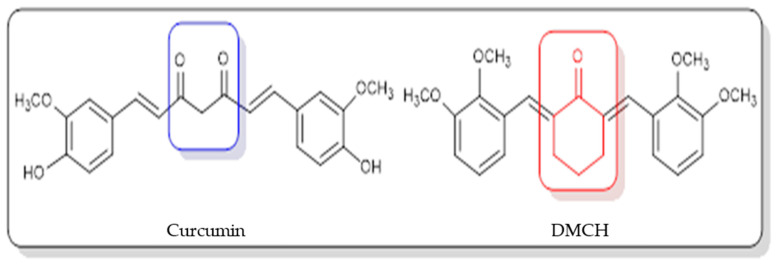
Chemical structures of curcumin and (2E,6E)-2,6-bis(2,3-dimethoxybenzylidene) cyclohexanone (DMCH).

The incidence of colorectal or colon cancer has been progressively increasing in recent years, and it has become the third most common type of cancer worldwide and a leading cause of cancer death [1]. According to the Malaysian National Cancer Registry Report 2012–2016 [2], colorectal cancer was the second most common cancer in Malaysia after breast cancer. It was also reported that this type of cancer was the second most common cancer to cause death and that it had been diagnosed in both males and females. The type of treatment that is recommended depends on the stage of cancer. The treatments that can be applied are laparoscopic colectomy [3], radiation, and chemotherapy, but these treatments may have side effects for the patients such as loss of appetite, alopecia (hair loss), constipation, and vomiting [4]. One of the alternative ways to fight cancer is by using natural products [5]. A natural product refers to a compound derived from natural sources such as plants, animals, or microorganisms. Discoveries of natural products in pharmacology and therapeutics have paved an alternative way to treat cancer, as it was found that the most useful sources possessing anticancer effects are compounds isolated from plants [6]. Curcumin derived from the rhizomes of *Curcuma longa* has been reported to have anticarcinogenic, antimalarial, antioxidant, antimutagenic, antibacterial, antiangiogenic, and anti-inflammatory properties [7]. Even though curcumin has been studied for decades, due to its low solubility, its usage as a therapeutic agent has been compromised [8]. In statistics, it was reported that there were new drugs formulated showing poor water solubility, which becomes the limiting factor in the absorption of the drug after oral admission [9]. Thus, efforts to identify potential solutions or alternatives that can modify the natural compound structure to achieve better selectivity against cancer cell lines are required. Although there are several reports that have indicated curcumin as a potential candidate for future drug discovery, bioavailability issues such as poor absorption, rapid metabolism, low serum levels, and limited tissue distribution remain a concern for scientific communities to resolve [8,9]. Structural modification of the parent compound is one of the best ways to improve the drug efficacy and to control the bioavailability-related issues. For example, a curcumin derivative, 2,6-bis(4-hydroxy-3-methoxybenzylidene) cyclohexanone (BHMC), has been synthesized and reported to possess potential cytotoxicity and antinociceptive attributes comparable to curcumin [10,11,12]. A study on BHMC demonstrated that it showed a lower IC_50_ value in cytotoxicity and effectively displayed in vivo antitumor effects [10]. In this study, (2E,6E)-2,6-bis(2,3-dimethoxybenzylidene) cyclohexanone (DMCH) (Figure 1) was synthesized, and the apoptotic mechanism of DMCH towards the human colon cancer cell lines HT29 and SW620 was investigated in detail.

## 2. Results

### 2.1. DMCH Inhibits the Cell Proliferation of SW620 and HT29

Results from the MTT assay demonstrated that DMCH could inhibit the proliferation of SW620 and HT29. The cells were treated for three time periods, which were 24 h, 48 h, and 72 h. Table 1 shows the value of inhibitory concentration (IC_50_) of DMCH towards both cell lines. After 24 h, both cells displayed IC_50_ values exceeding 30 µM, while the IC_50_ values obtained after 48 and 72 h for SW620 cells were 7.500 ± 1.185 and 5.000 ± 0.157 µM, respectively, as displayed in Table 1. Meanwhile, the IC_50_ values after 48 and 72 h for HT29 cells were 9.80 ± 0.553 and 8.20 ± 0.761 µM, respectively. There were no IC_50_ values observed for splenocytes after 24, 48, or 72 h of treatment.

### 2.2. DMCH Alters the Morphology and Induces Apoptosis in SW620 and HT29

**Figure 2:**
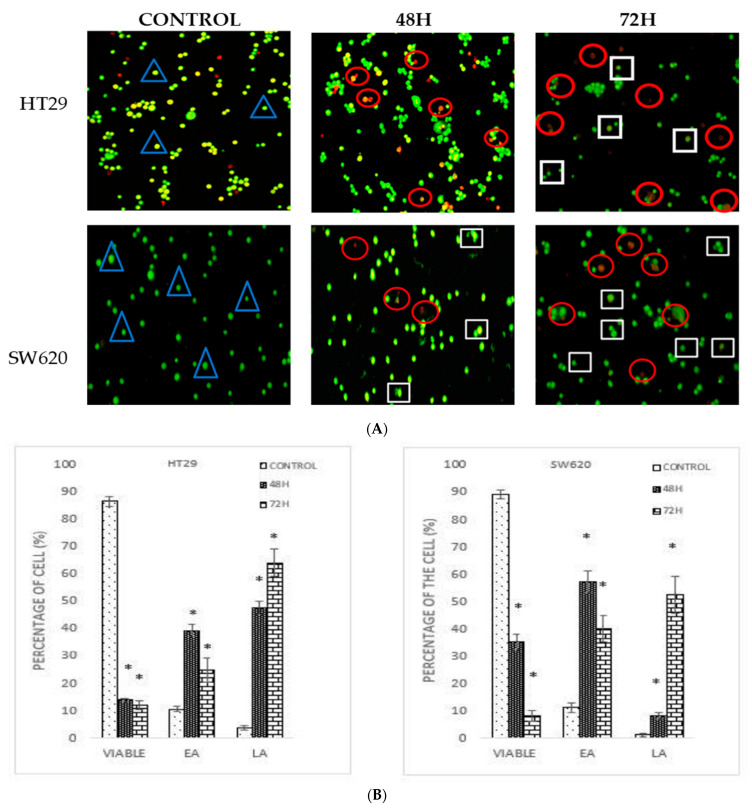
(**A**) Morphological changes in HT29 and SW620 after 48 h and 72 h treatment with DMCH. (**B**) Quantification analysis of HT29 and SW620 based on the cell uptake of acridine orange and propidium iodide (blue triangle: viable; white square: early apoptosis; red circle: late apoptosis/necrosis). EA (early apoptosis), LA (late apoptosis). All data are expressed as mean ± standard error mean (S.E.M.). * *p* < 0.05 compared with the corresponding controls.

Acridine orange/propidium iodide (AO/PI) double staining assay was conducted to examine the cell death of colon cancer cells microscopically. Viable cells are stained green colour [13], while the green stained cells showing some distinct apoptotic features such as membrane blebbing and chromatin condensation are early apoptotic cells. The cells that appear orange are the late apoptotic cells, whilst necrotic cells are stained red [14]. Based on the MTT assay, two different inhibition concentrations (IC_50_ at 48- and 72-h time periods) of DMCH were used to treat both SW620 and HT29 for the AO/PI assay. Figure 2 shows the stained SW620 and HT29 cells that were treated with DMCH for the two time periods. Based on Figure 2A, different morphological changes were observed on the DMCH-treated cells. As the incubation time increased, the proportion of SW620 and HT29 cells that exhibited early apoptosis and late apoptosis was significantly higher (*p* < 0.05) as shown in Figure 2B. The results revealed a significant (*p* < 0.05) progression in apoptosis over the exposure time upon DMCH treatment in both cell lines.

### 2.3. DMCH Induces Apoptotic Cell Death in Colon Cancer Cells HT29 and SW620

**Figure 3:**
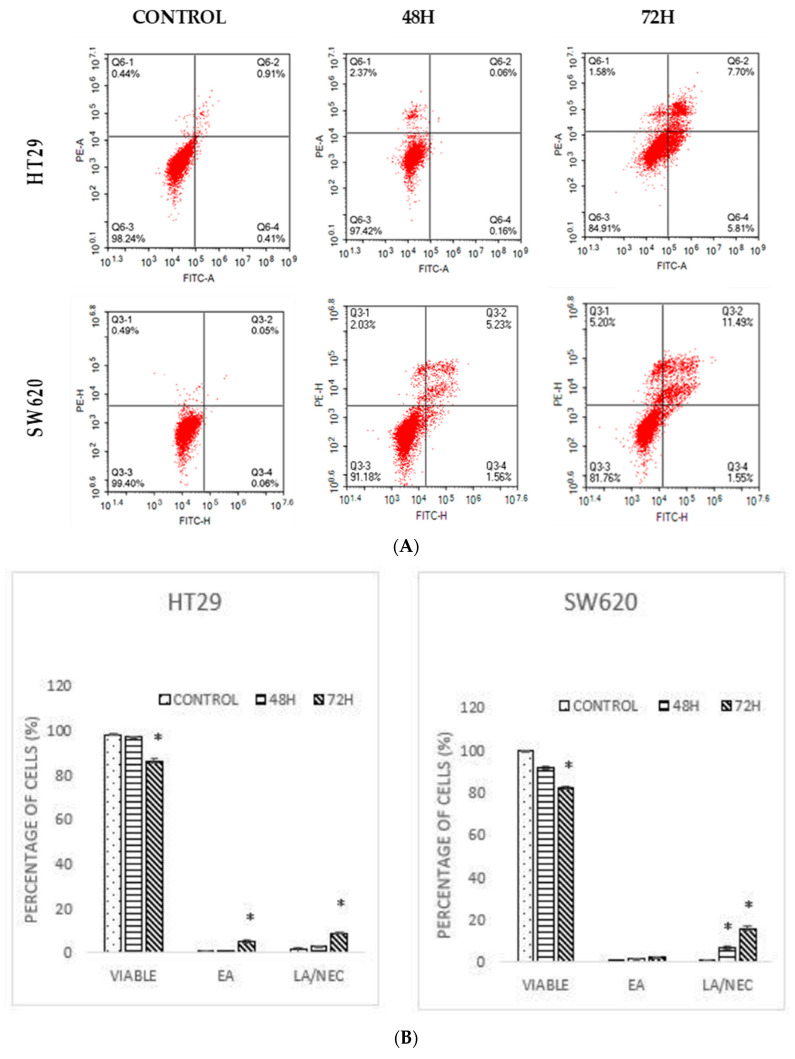
(**A**) A dot plot of Annexin V/FITC of HT29 and SW620 after treatment with DMCH for two time periods (48 h and 72 h). (**B**) Quantification of Annexin V/FITC of HT-29 and SW620. EA (early apoptosis), LA/NEC (late apoptosis/necrosis). All data are expressed as mean ± standard error mean (S.E.M.). * *p* < 0.05 compared with corresponding controls.

Annexin V/FITC is an assay to observe the apoptotic population [15]. According to the result displayed in Figure 3, a high population of viable cells was observed in the untreated group for HT29 (98.18 ± 0.25%) and SW620 (99.38 ± 0.14%) cells. A pattern of the cell population shifting from viable cells to early apoptotic to late apoptotic was observed for both cell lines for both time periods (Figure 3A). However, a significant increment in the early and late apoptotic/necrotic cells was observed after 72 h for both HT29 (8.83 ± 0.42%) and SW620 (15.70 ± 1.03%) cells (Figure 3). The results revealed that DMCH induced apoptosis and necrosis in a time-dependent manner.

### 2.4. DMCH Induces Cell Cycle Accumulation at Sub-G0/G1 in HT29 and SW620

**Figure 4:**
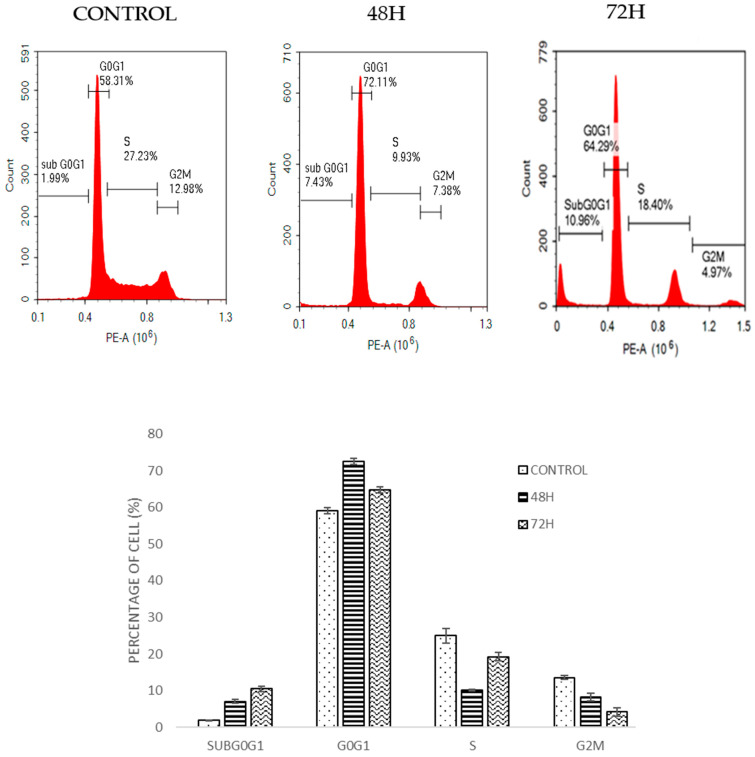
Cell cycle histogram and bar chart analysis for HT-29 after being treated with DMCH for two time periods (48 h and 72 h). All data are expressed as mean ± standard error mean (S.E.M.).

**Figure 5:**
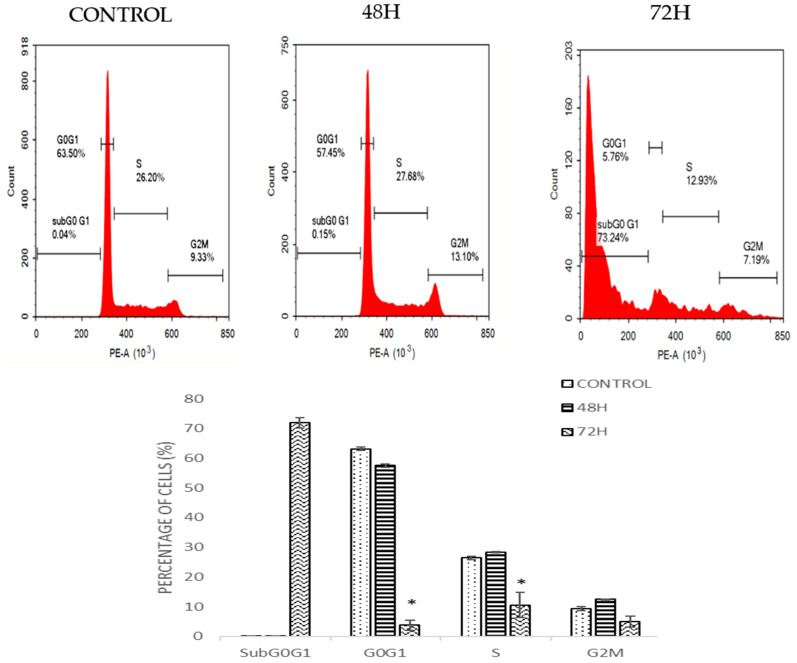
Cell cycle histogram and bar chart analysis for SW620 after being treated with DMCH for two time periods (48 h and 72 h). All data are expressed as mean ± standard error mean (S.E.M.). * *p* < 0.05 compared with corresponding controls.

Cell cycle analysis by flow cytometry was used to determine the effect of DMCH on cell cycle progression in both SW620 and HT29 cells after 48 and 72 h. As shown in Figure 4, there was a slight increase in the cell percentage at sub-G0/G1 phase for HT29 as the time increased. For SW620 cells, DMCH treatment resulted in a significant increase in the sub-G0/G1 phase (*p* < 0.05) where it increased from 0.15 ± 0.05% at 48 h to 73.24 ± 1.65% at 72 h (Figure 5). Meanwhile, comparatively, the percentage of cells in the sub-G0/G1 phase at 72 h for HT29 cells was only 10.96± 0.94%.

### 2.5. DMCH Induces Changes in the Mitochondrial Membrane Potential in SW620 and HT29

**Figure 6:**
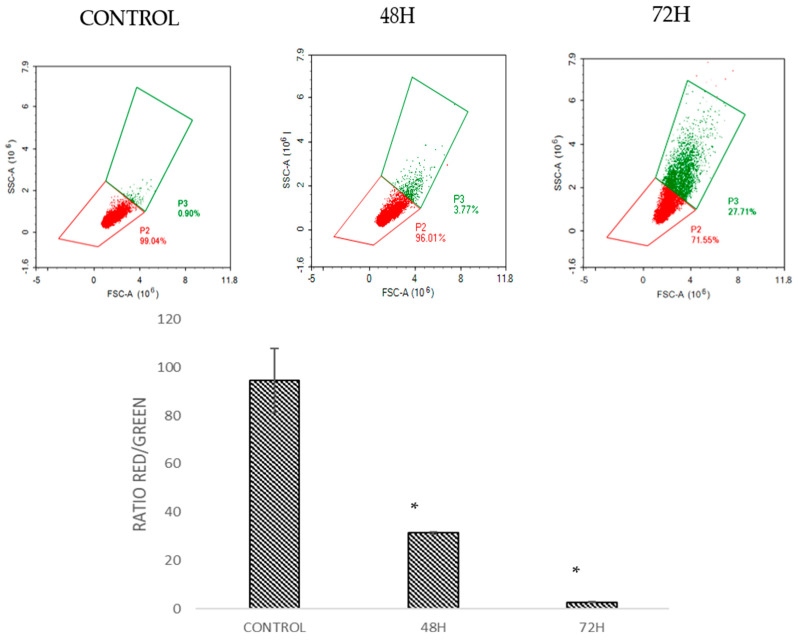
Histogram and quantification analysis of the depolarization of mitochondrial membrane potential for HT29 after being treated with DMCH for 48 h and 72 h. All data are expressed as mean ± standard error mean (S.E.M.). * *p* < 0.05 compared with corresponding controls.

**Figure 7:**
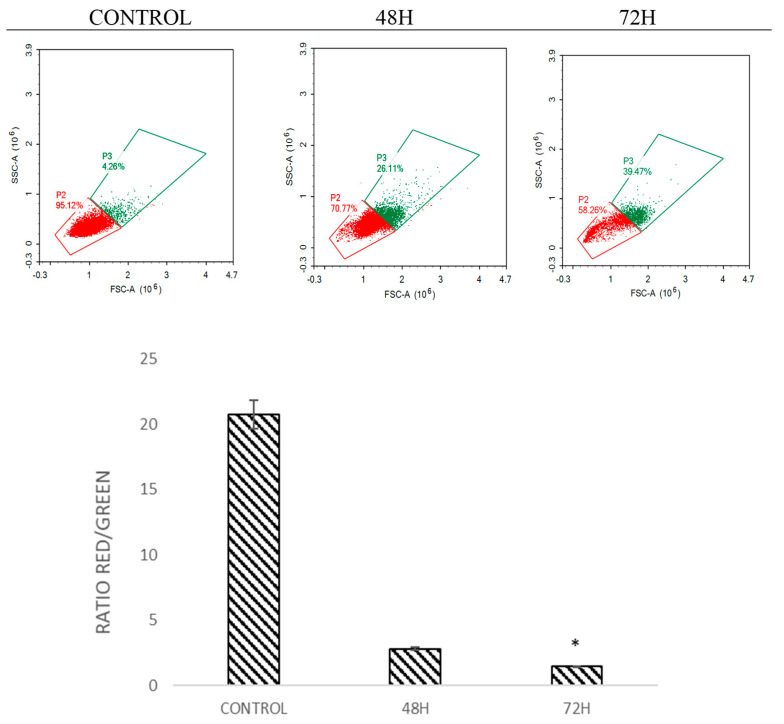
Histogram and quantification analysis of the depolarization of mitochondrial membrane potential for SW620 after being treated with DMCH for 48 h and 72 h. All data are expressed as mean ± standard error mean (S.E.M.). * *p* < 0.05 compared with corresponding controls.

The mitochondrial membrane potential was analysed by staining the cells with JC-1 dye [16]. This dye is a marker that detects the loss of mitochondrial membrane potential [17]. The dye will emit green and red fluorescence in response to mitochondrial depolarization. The JC-1 dye present in healthy cells in the form of aggregates emits red fluorescence, while in apoptotic cells the dye is in monomeric form and emits green fluorescence [15]. The ratio of red to green was analysed. Histogram analysis in Figure 6 and Figure 7 shows the ratio of red/green fluorescence after treatment with DMCH in HT-29 and SW620 for 48 and 72 h. As illustrated in Figure 6 and Figure 7, as the time of treatment increased, the ratio of monomers observed was higher which indicates that apoptosis was induced in HT29 and SW620 cells in a time-dependent manner.

### 2.6. DMCH Regulates Several Apoptosis-Related Genes and Proteins

**Figure 8:**
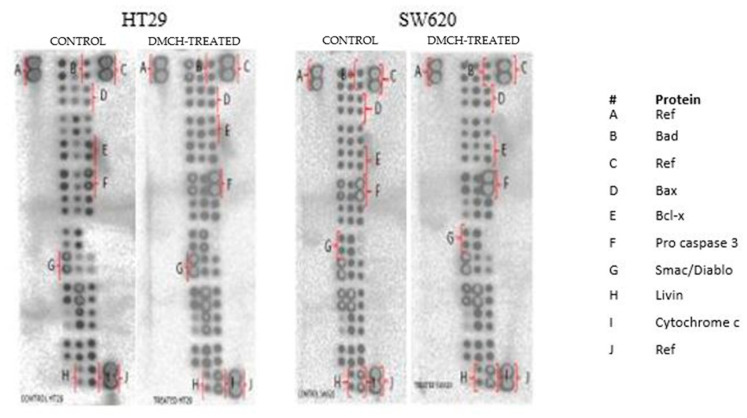
The proteome profiler membranes depicting expression of the regulated proteins between control and DMCH-treated HT29 and SW620.

The effects of apoptotic proteins in both HT29 and SW620 treated with DMCH were demonstrated through use of a human apoptosis proteome profiler. A depiction of regulated proteins is displayed in Figure 8, while the levels of regulation of the proteins are expressed in Table 2. Among the apoptosis-related proteins, the upregulation of Bax and Bad proteins and the downregulation of Livin proteins were observable in both HT29 and SW620 cell lines. However, both cell lines gave different trends in the regulation of Bcl x, pro-caspase 3, cytochrome c, and SMAC/Diablo proteins.

## 3. Discussion

In this study, it was found that DMCH exhibited cytotoxic activity in human colonic adenocarcinoma cell lines HT29 and SW620. HT29 and SW620 were chosen as both have different characteristics, where SW620 was reported to be highly metastatic when compared with HT29, while HT-29 cells are more adhesive than other colon cancer cells. DMCH inhibited the proliferation of both HT29 and SW620 in a time-dependent manner. Based on the inhibitory concentration (IC_50_) value of the compound, both cells demonstrated different efficiencies, respectively. Based on the IC_50_ value, DMCH inhibited proliferation better in SW620 compared with HT29. Furthermore, DMCH treatment showed no adverse effect on normal cells as represented by splenocytes when given the same dosage and exposure time as that used on HT29 and SW620 cell lines. This suggests that the compound is not cytotoxic to non-cancerous cells. DMCH is believed to have better solubility and effectiveness compared with natural curcumin, which required a higher concentration to exhibit inhibitory concentrations as indicated in Table 1. In this study, a low DMCH concentration of 9.8 µM was required to achieve 50% inhibition in HT29 cells, and only 7.5 µM for SW620 at 48 h while curcumin required an almost three times higher concentration to achieve similar results. A similar trend was displayed in a previous study which found that against the HT29 and SW620 cell lines, treatment of curcumin required a higher concentration to obtain the IC_50_ value compared with the curcumin analogue they studied [18]. Preliminary MTT screening done on breast cancer lines and osteosarcoma cell lines showed that treatment with curcumin analogues showed a better cytotoxic effect in a concentration- and time-dependent manner, as compared to natural curcumin [7,12]. Apoptosis is one of the most crucial factors in the development of anticancer therapeutic agents, and the induction of apoptosis can be evaluated by morphological and biochemical assessments [4]. From the AO/PI assay, it was observed that both HT29 and SW620 cells underwent apoptosis as shown by the results of DMCH treatment. Apoptotic cells can be categorized based on their morphological features such as chromatin condensation, cell shrinkage, and membrane blebbing [8,13]. During early apoptosis, cells will appear rounded in shape and have a mass with dark eosinophilic cytoplasm and dense purple nuclear chromatin fragment. Cells undergo shrinkage resulting in a smaller size, and the cytoplasm is denser and tightly packed [19]. The population of cells undergoing apoptosis increased as time increased. In the Annexin V/FITC assay, there was a shift in the pattern for the externalization of phosphatidylserine in the DMCH-treated cells for both cell lines. Increasing percentages of apoptotic cells were observed in 48 h and 72 h treatments when compared with the control treatment. This shows that DMCH possesses the capability to induce apoptosis in a time-dependent manner in both SW620 and HT29 cells.

The cell cycle is the set of events that is responsible for the duplication of the cell, where each checkpoint plays a significant role. From the cell cycle analysis (Figure 4), there was a relative increase in sub-G0/G1 peak in a time-dependent manner in both cell lines. Accumulation at this phase indicates that the cells were undergoing programmed cell death via apoptosis [20]. The results of this cellular mechanism study show that DMCH affected the cell cycle machinery as it exerts its cytotoxic effect on HT29 and SW620 cells by inducing apoptosis. The accumulation at sub-G0/G1 phase was much higher in SW620 cells compared with HT29 cells, which allows us to conclude that DMCH is more potent as an apoptosis inducer in SW620 cells compared with HT29 cells. 

There are two main pathways involved in apoptosis, which are the intrinsic and extrinsic pathways [19,21]. The intrinsic pathway is highly dependent on mitochondrial-related proteins while the extrinsic pathway, which is also known as the death receptor pathway, involves transmembrane receptor-mediated interactions [19]. As demonstrated in the JC-1 assay, DMCH was capable of triggering the depolarization of the mitochondrial membrane potential in both HT29 and SW620, proved by the significant decrease (*p* < 0.05) in the ratio of red/green in the treated samples compared with the control [14]. This event can be proved by the expression of several proteins examined via proteome profiler analysis (Figure 8). According to Table 2, DMCH was able to upregulate the pro-apoptotic proteins Bad and Bax in both HT29 and SW620 cells and downregulate the expression of antiapoptotic protein Bcl x in HT29. These proteins are members of the Bcl-2 protein family, which is heavily involved in intrinsic apoptosis events and controls the membrane integrity of mitochondria and the cytochrome c release process [22]. However, upregulated expression of pro-caspase 3, cytochrome c, and SMAC/Diablo was only observable in HT29 and not in SW620, where these proteins did not show any significant changes in expression. Furthermore, both cell lines showed a downregulation of Livin protein expression. Livin is a novel inhibitor of apoptosis as it inhibits the activation of caspase-9 [22]. The activation of caspase 9 is important as it will activate caspase 3, which promotes nucleus fragmentation and disruption of the nucleus membrane [22]. Half of the apoptotic genes studied were reversely regulated in SW620 cells when compared with HT29, and this may be caused by different resistance and selectivity of DMCH treatment towards different cell lines. Overall results showed that DMCH displayed greater potency towards SW620 compared with HT29, indicating that it is more effective on metastasized cells. 

## 4. Materials and Methods

### 4.1. Synthesis of (2E,6E)-2,6-Bis(2,3-Dimethoxybenzylidene) Cyclohexanone (DMCH)

DMCH was chemically synthesized from 2,3-dimethoxybenzaldehyde and cyclohexanone at our laboratory using recently published protocols [12]. The structure was determined by 1H NMR and EI-MS mass spectrometry. The synthetic DMCH was purified by repeated column chromatography and crystallization.

### 4.2. Cell Culture

The colon cancer cell lines HT-29 and SW620 were purchased from the American Type Culture Collection (ATCC, Manassas, VA, USA). SW620 cells were maintained in Dulbecco’s Modified Eagle Medium (DMEM) while HT29 cells were maintained in Roswell Park Memorial Institute (RPMI) medium. Both media were supplemented with 10% fetal bovine serum (PAA, Austria) and 1% penicillin-streptomycin (Gibco, Thermo Fisher Scientific, Waltham, MA, USA).

### 4.3. Cell Viability Assay (MTT Assay) 

Both SW620 and HT29 cells were grown in an incubator at 37 °C under 5% CO_2_ condition with their respective media. After the cells were harvested, they were counted and seeded at 1 × 10^5^ cells per well in a 96-well plate overnight before being treated with DMCH. The following day, cells were treated with various concentrations of DMCH. Then, the treated cells were incubated for different time periods, which were 48 h and 72 h. Following incubation for the designated time period, 20 μL of (5 mg/mL) MTT 3-[4, 5-dimethylthiazol-2-yl]-2, 5 diphenyltetrazolium bromide (Merck, Kenilworth, NJ, USA) reagent was added into each well and then cells were incubated again for another 3 to 4 h. Next, the solution was removed and 100 μL of dimethyl sulfoxide (DMSO) was added into the wells. Afterward, the plate was read at 575 nm using a microtiter plate reader (μQuant, Bio-Tek Instrument, Winooski, VT, USA). The results were analysed as the percentage proliferation of the cells regarding the concentration of the sample treated. The following formula was used to determine the percentage of viable cells:

Percentage of viability (%) = (Absorbance of sample at 570 nm)/(Absorbance of control at 570 nm) × 100.

### 4.4. Cell Treatment

Based on the result from the MTT assay (Table 1), doses were based on the values of IC_50_ for different time periods (48 h and 72 h) in SW620 and HT29.

### 4.5. Acridine Orange/Propidium Iodide (AO/PI) Double Staining Assay

Acridine orange/propidium iodide (AO/PI) double staining assay was performed on colon cancer cell lines to determine the mode of cell death microscopically. HT29 and SW620 cell lines were seeded in a 6-well plate at a density of 2 × 10^5^ cells/well. Then, cells were treated with DMCH sample according to 48 h and 72 h concentrations. The treatment-free culture was used as a negative control, and cells were detached and collected at the end of each incubation time. Then, cells were washed with PBS and incubated with a 1:1 ratio of acridine orange (10 µg/mL) and propidium iodide (10 µg/mL). An amount of 10 µL of incubated suspension cells was placed on a slide and viewed immediately under a fluorescent microscope (Nikon FC-35DX, Nikon, Tokyo, Japan).

### 4.6. Cell Cycle Analysis

The effect of the DMCH sample on the cell cycle of HT29 and SW620 cells was investigated using flow cytometry cell cycle analysis. Cell cycle assay was carried out using the CycleTEST Plus DNA Reagent Kit according to the instructions (BD Pharmingen, San Diego, CA, USA). The cells were seeded at a density of 2.0 × 10^5^ cells/well overnight. Then, cells were treated with different dosages of DMCH based on the concentration results of the MTT assay for 48 h and 72 h. After the treatment, cells were collected, and the pellet was resuspended in 200 μL of trypsin (solution A) and RNAse (solution B) for 10 min each followed by 250 μL of propidium iodide (solution C) for 15 min. After that, flow cytometric analysis was performed within 3 h using a Novocytes flow cytometer (ACEA Biosciences Inc., San Diego, CA, USA).

### 4.7. Annexin V/FITC Assay

The apoptosis effect on HT29 and SW620 cells was studied using an Annexin V fluorescence kit (BD Pharmingen, San Diego, CA, USA) according to the manufacturer’s instructions without any modifications. Briefly, cells were seeded for 24 h at a density of 2.0 × 10^5^ cells per well in a 6-well plate. Then, the seeded cells were treated with the designated concentrations for 48 h and 72 h. After the 48 h (or 72 h) of treatment, the treated cells were harvested and collected as a pellet. Next, the pellet was resuspended in 400 μL of binding buffer and stained with 5 μL of FITC-Annexin-V and 5 μL of PI provided in the kit. Afterward, the cells were analysed using a Novocyctes flow cytometer (ACEA Biosciences Inc., San Diego, CA, USA).

### 4.8. JC-1 (Mitoscreen) Assay

JC-1 assay was used to detect the depolarization of the mitochondrial membrane potential of the cell after being treated with the selected doses of DMCH for 48 h. This assay was accomplished using a BD MitoScreen Kit (BD Pharmingen, San Diego, CA, USA). In brief, the cells were seeded in a 6-well plate for 24 h at a density of 2.0 × 10^5^ cells per well. Then, the cells were treated with various concentrations as described in Table 1. This assay was performed based on the manufacturer’s instructions provided with the kit without any modification. After 48 h of incubation, the cells were harvested, collected, and incubated with 500 μL of JC-1 stock solution for 15 min. Then, analysis was performed using a Novocyctes flow cytometer (ACEA Biosciences Inc., San Diego, CA, USA).

### 4.9. Proteome Profiler Angiogenesis

Colon cancer cell lines SW620 and HT29 that had been treated with DMCH for 48 h were investigated for the expression profiles of apoptosis-related proteins. This was conducted using the Human Apoptosis Array Kit (R&D Systems, Minneapolis, MN, USA). Firstly, the proteins from both cells were extracted using 600 µL of RIPA buffer (50 mM Tris-HCl, 150 mM NaCl, 1.0% TritonX-100, 0.5% sodium deoxycholate, 0.1% SDS) supplemented with 10 mg of pre-made protease inhibitor cocktails (Roche, Mississauga, ON, Canada). After that, the proteins were quantified using the Bradford assay (Sigma, St. Louis, MO, USA). Then, membranes were blocked by blocking buffer for an hour. At the same time, the proteins were prepared with the addition of 1 mL Lysis Buffer 17 to the protein lysate. The prepared protein was loaded onto the membrane and was incubated at 4 °C overnight. The following day, the membranes were washed three times with 20 mL of wash buffer provided in the kit. Next, the membrane was transferred into a 4-well multi-dish that contains the reconstituted detection antibody cocktails, and it was incubated for an hour on a shaker. Then, the membrane was again washed with a washing buffer three times. Two millilitres of Streptavidin-HRP was added onto the membrane followed by incubation for 30 min. Then, the membrane was washed three times. Afterward, the membrane was loaded with 1.0 mL of Chemi Reagent Mix and then the membrane was scanned using ChemiDoc XRS (Bio-Rad, Hercules, CA, USA).

### 4.10. Statistical Analysis

All experiments were performed in triplicate, and average values were obtained. The statistical analysis was performed using GraphPad Prism. For the experimental analysis, one-way ANOVA was performed using Tukey’s post-hoc test. The significance was set at *p* < 0.05. The comparison of statistical significance was done between control HT29/SW620 (untreated) and HT-29/SW-620 treated groups.

## 5. Conclusions

DMCH induced apoptosis in both HT29 and SW620 in a time-dependent manner. This study also showed that DMCH induced sub-G0/G1 arrest in both cell lines, and the regulation in pro-apoptotic proteins indicates that cells underwent programmed cell death via apoptosis when treated with DMCH. Overall, this study shows that DMCH is a potent cytotoxic agent on colon cancer cell lines, with potential drug candidate activity.

## Figures and Tables

**Table 1:** The inhibitory concentration (IC_50_) value of DMCH on colon cancer cell lines (SW620 and HT29) after 24, 48, and 72 h of treatment. Cytotoxicity effects of DMCH on colon cancer cell lines.

Cells	HT29			SW620			Splenocyctes		
Time	24 h	48 h	72 h	24 h	48 h	72 h	24 h	48 h	72 h
**IC_50_ of DMCH (μM)**	>30	9.800 ± 0.553	8.200 ± 0.761	>30	7.500 ± 1.185	5.000 ± 0.157	>30	>30	>30
**IC_50_ of curcumin (μM)**	>30	26.700 ± 1.527	20.660 ± 1.155	>30	18.630 ± 0.321	15.660 ± 1.524	>30	>30	>30

Notes: The IC_50_ values of DMCH and curcumin in SW620 and HT29 after 24 h, 48 h, and 72 h of treatment. The IC_50_ value for splenocytes after 24 h, 48 h, and 72 h of treatment. All experiments were performed in triplicate. All treatments are expressed as mean ± standard error mean (S.E.M.).

**Table 2:** Apoptosis pathway-related protein expression in DMCH-treated HT29 and SW620.

Cell Lines	Proteins	Relative Intensity (Fold Change)	Regulation
HT29	Bad	1.100 * ± 0.130	Up
	Bax	1.200 * ± 0.060	Up
	Bcl x	−0.700 * ± 0.130	Down
	Pro caspase 3	2.100 * ± 0.180	Up
	Cytochrome c	1.300 * ± 0.003	Up
	Livin	−0.900 * ± 0.090	Down
	SMAC/Diablo	1.500 * ± 0.050	Up
SW620	Bad	1.100 * ± 0.005	Up
	Bax	1.100 * ± 0.600	Up
	Bcl x	1.200 * ± 0.060	Up
	Pro caspase 3	−0.200 * ± 0.030	Down
	Cytochrome c	−0.900 ± 0.006	Down
	Livin	−0.900 ± 0.040	Down
	SMAC/Diablo	−0.900 ± 0.070	Down

Note: Human apoptosis proteome profiler of HT29 and SW620 colon cancer cell lines treated with DMCH (IC50) for 48 h. All data are expressed as mean ± SD. The (−) symbol indicates downregulated protein expression. * *p* < 0.05 compared with corresponding controls.

## Data Availability

Not applicable.

## Sample Availability

Samples of the DMCH are available from the authors.

## References

[1] De Rosa M., Rega D., Costabile V., Duraturo F., Niglio A., Izzo P., Pace U., Delrio P. (2016). The biological complexity of colorectal cancer: Insights into biomarkers for early detection and personalized care. Ther. Adv. Gastroenterol..

[2] Aziah A.M., Hashimah B., Nirmal K., Siti Zubaidah A.R., Puteri N.A., Nabihah A., Sukumaran R., Balqis B., Nadia S.M.R., Sharifah S.S.S. (2019). Malaysia National Cancer Registry Report (MNCR) 2012–2016. Natl. Cancer Registry NCI.

[3] Ahad S., Figueredo E.J. (2007). Laparoscopic Colectomy. Medscape Gen. Med..

[4] Abidin S.A.Z., Rajadurai P., Chowdhury E.H., Othman I., Naidu R. (2018). Cytotoxic, Anti-Proliferative and Apoptosis Activity of l-Amino Acid Oxidase from Malaysian Cryptelytrops purpureomaculatus (CP-LAAO) Venom on Human Colon Cancer Cells. Molecules.

[5] Nobili S., Lippi D., Witort E., Donnini M., Bausi L., Mini E., Capaccioli S. (2009). Natural compounds for cancer treatment and prevention. Pharmacol. Res..

[6] Dholwani K.K., Saluja A.K., Gupta A.R., Shah D.R. (2008). A review on plant-derived natural products and their analogs with anti-tumor activity. Indian J. Pharmacol..

[7] Ali N.M., Yeap S.K., Abu N., Lim K.L., Ky H., Pauzi A.Z.M., Ho W.Y., Tan S.W., Alan-Ong H.K., Zareen S. (2017). Synthetic curcumin derivative DK1 possessed G2/M arrest and induced apoptosis through accumulation of intracellular ROS in MCF-7 breast cancer cells. Cancer Cell Int..

[8] Zhang X., Tian Y., Li Z., Tian X., Sun H., Liu H., Moore A., Ran C. (2013). Design and Synthesis of Curcumin Analogues for in Vivo Fluorescence Imaging and Inhibiting Copper-Induced Cross-Linking of Amyloid Beta Species in Alzheimer’s Disease. J. Am. Chem. Soc..

[9] Srinivasan M. (1972). Effect of curcumin on blood sugar as seen in a diabetic subject. Indian J. Med. Sci..

[10] Ming-Tatt L., Khalivulla S.I., Akhtar M.N., Mohamad A.S., Perimal E.K., Khalid M.H., Akira A., Lajis N.H., Israf D.S., Sulaiman M.R. (2012). Antinociceptive activity of a synthetic curcuminoid analogue, 2,6-bis-(4-hydroxy-3- methoxybenzylidene) cyclohexanone, on nociception-induced models in mice. Basic Clin. Pharmacol. Toxicol..

[11] Razak N.A., Akhtar M.N., Abu N., Ho W.Y., Tan S.W., Zareen S., Tajuddin S.N., Long K., Yeap S.K., Alitheen N.B. (2017). In vivo anti-tumor effect of curcumin derivative(2E,6E)-2,6-bis(4-hydroxy-3-methoxybenzylidene) cyclohexanone (BHMC) on 4T1 breast cancer cells. RSC Adv..

[12] Zamrus S.N.H., Akhtar M.N., Yeap S.K., Quah C.K., Loh W.-S., Alitheen N.B., Zareen S., Tajuddin S.N., Hussin Y., Shah S.A.A. (2018). Design, synthesis and cytotoxic effects of curcuminoids on HeLa, K562, MCF-7 and MDA-MB-231 cancer cell lines. Chem. Cent. J..

[13] Baharara J., Namvar F., Ramezani T., Mousavi M., Mohamad R. (2015). Silver Nanoparticles Biosynthesized Using Achillea biebersteinii Flower Extract: Apoptosis Induction in MCF-7 Cells via Caspase Activation and Regulation of Bax and Bcl-2 Gene Expression. Molecules.

[14] Aziz M.N.M., Hussin Y., Rahim N.F.C., Nordin N., Mohamad N.E., Yeap S.K., Yong C.Y., Masarudin M.J., Cheah Y.K., Abu N. (2018). Curcumin Analog DK1 Induces Apoptosis in Human Osteosarcoma Cells In Vitro through Mitochondria-Dependent Signaling Pathway. Molecules.

[15] Zhao Z., Yang Y., Liu W., Li Z. (2018). T59, a New Compound Reconstructed from Curcumin, Induces Cell Apoptosis through Reactive Oxygen Species Activation in Human Lung Cancer Cells. Molecules.

[16] Xue C., Pasolli H.A., Piscopo I., Gros D.J., Liu C., Chen Y., Chiao J.W. (2014). Mitochondrial structure alteration in human prostate cancer cells upon initial interaction with a chemo-preventive agent phenethyl isothiocyanate. Cancer Cell Int..

[17] Chakravarthi B.V., Sujay R., Kuriakose G.C., Karande A.A., Jayabaskaran C. (2013). Inhibition of cancer cell proliferation and apoptosis-inducing activity of fungal Taxol and its precursor baccatin III purified from endophytic Fusarium solani. Cancer Cell Int..

[18] Hussin Y., Aziz M.N.M., Rahim N.F.C., Yeap S.K., Mohamad N.E., Masarudin M.J., Nordin N., Rahman N.M.A.-N.A., Yong C.Y., Akhtar M.N. (2018). DK1 Induces Apoptosis via Mitochondria-Dependent Signaling Pathway in Human Colon Carcinoma Cell Lines In Vitro. Int. J. Mol. Sci..

[19] Elmore S. (2007). Apoptosis: A Review of Programmed Cell Death. Toxicol. Pathol..

[20] Karimian H., Mohan S., Moghadamtousi S.Z., Fadaeinasab M., Razavi M., Arya A., Kamalidehghan B., Ali H.M., Noordin M.I. (2014). *Tanacetum polycephalum* (L.) Schultz-Bip. Induces Mitochondrial-Mediated Apoptosis and Inhibits Migration and Invasion in MCF7 Cells. Molecules.

[21] Kiraz Y., Adan A., Yandim M.K., Baran Y. (2016). Major apoptotic mechanisms and genes involved in apoptosis. Tumor Biol..

[22] Kasof G.M., Gomes B.C. (2001). Livin, a Novel Inhibitor of Apoptosis Protein Family Member. J. Biol. Chem..

